# A strawman with machine learning for a brain: A response to Biedermann (2022) the strange persistence of (source) “identification” claims in forensic literature

**DOI:** 10.1016/j.fsisyn.2022.100230

**Published:** 2022-05-06

**Authors:** Geoffrey Stewart Morrison, Daniel Ramos, Rolf JF Ypma, Nabanita Basu, Kim de Bie, Ewald Enzinger, Zeno Geradts, Didier Meuwly, David van der Vloed, Peter Vergeer, Philip Weber

**Affiliations:** Forensic Data Science Laboratory, Aston University, Birmingham, UK; Forensic Evaluation Ltd, Birmingham, UK; AUDIAS – Audio, Data Intelligence and Speech, Escuela Politécnica Superior, Universidad Autónoma de Madrid, Madrid, Spain; Netherlands Forensic Institute, The Hague, the Netherlands; Forensic Data Science Laboratory, Aston University, Birmingham, UK; Forensic Data Science Laboratory, Aston University, Birmingham, UK; Netherlands Forensic Institute, The Hague, the Netherlands; Eduworks Corporation, Corvallis, OR, USA; Forensic Data Science Laboratory, Aston University, Birmingham, UK; Netherlands Forensic Institute, The Hague, the Netherlands; University of Amsterdam, Amsterdam, the Netherlands; Netherlands Forensic Institute, The Hague, the Netherlands; University of Twente, Enschede, the Netherlands; Netherlands Forensic Institute, The Hague, the Netherlands; Netherlands Forensic Institute, The Hague, the Netherlands; Forensic Data Science Laboratory, Aston University, Birmingham, UK

**Keywords:** Forensic inference, Machine learning

## Abstract

We agree wholeheartedly with Biedermann (2022) FSI Synergy article 100222 in its criticism of research publications that treat forensic inference in source attribution as an “identification” or “individualization” task. We disagree, however, with its criticism of the use of machine learning for forensic inference. The argument it makes is a strawman argument. There is a growing body of literature on the calculation of well-calibrated likelihood ratios using machine-learning methods and relevant data, and on the validation under casework conditions of such machine-learning-based systems.

Letter to Editor:

Biedermann [1] is critical of research publications that treat forensic inference in source attribution as an “identification” or “individualization” task. Biedermann [1] argues that such publications condone unscientific attitudes and practices, foster unrealistic expectations among consumers of forensic science, and undermine trust in peer-reviewed publications because so-called “original research papers” are not, in fact, well grounded. With respect to these points, we agree wholeheartedly with Biedermann [1].

With respect to criticism of machine learning, however, we feel that Biedermann [1] makes a strawman argument. It defines “standard” machine learning as outputting categorical decisions and then criticizes the use of “standard” machine learning for forensic inference because it outputs categorical decisions. There are indeed research publications that misapply machine learning to forensic-inference problems, including using algorithms that output categorical decisions, e.g. [2]. But we fear that many readers will get the impression from Biedermann [1] that this is the only way (or at least the primary way) that machine learning is applied to forensic inference. There is in fact a growing body of literature on the calculation of well-calibrated likelihood ratios using machine-learning methods and relevant data, and on the validation under casework conditions of such machine-learning-based systems. Recent examples include [[3], [4], [5], [6], [7], [8], [9], [10], [11]].

## Disclaimer

All opinions expressed in the present paper are those of the authors, and, unless explicitly stated otherwise, should not be construed as representing the policies or positions of any organizations with which the authors are associated.

## Declaration of competing interest

The authors declare that they have no known competing financial interests or personal relationships that could have appeared to influence the work reported in this paper.

## Author contributions

**Morrison, Ramos, Ypma:** Writing - Original Draft, Writing - Review & Editing. **All other authors:** Writing - Review & Editing.
